# Cancer diagnosis and treatment platform based on manganese-based nanomaterials

**DOI:** 10.3389/fbioe.2024.1363569

**Published:** 2024-03-01

**Authors:** Jia Fei, Yanyan Liu, Ya Zeng, Mingqi Yang, Shanshan Chen, Xiaobing Duan, Ligong Lu, Muhe Chen

**Affiliations:** ^1^ Guangdong Provincial Key Laboratory of Tumor Interventional Diagnosis and Treatment, Zhuhai Institute of Translational Medicine, Zhuhai Clinical Medical College of Jinan University, Zhuhai, Guangdong, China; ^2^ Zhuhai Clinical Medical College of Jinan University (Zhuhai People's Hospital), Zhuhai, China

**Keywords:** cancer diagnosis, cancer treatment, tumor, TME (tumor microenvironment), manganese nanomaterials, nanomaterials

## Abstract

Cancer is a leading cause of death worldwide, and the development of new diagnostic and treatment methods is crucial. Manganese-based nanomaterials (MnNMs) have emerged as a focal point in the field of cancer diagnosis and treatment due to their multifunctional properties. These nanomaterials have been extensively explored as contrast agents for various imaging technologies such as magnetic resonance imaging (MRI), photoacoustic imaging (PAI), and near-infrared fluorescence imaging (NIR-FL). The use of these nanomaterials has significantly enhanced the contrast for precise tumor detection and localization. Moreover, MnNMs have shown responsiveness to the tumor microenvironment (TME), enabling innovative approaches to cancer treatment. This review provides an overview of the latest developments of MnNMs and their potential applications in tumor diagnosis and therapy. Finally, potential challenges and prospects of MnNMs in clinical applications are discussed. We believe that this review would serve as a valuable resource for guiding further research on the application of manganese nanomaterials in cancer diagnosis and treatment, addressing the current limitations, and proposing future research directions.

## 1 Introduction

Cancer, a major life-threatening disease, imposes a significant burden on global human health. Its incidence and mortality rates are rapidly rising, with an estimated 28.4 million new cancer cases projected by 2040 (Sung et al., 2021). A significant proportion of cancer cases are diagnosed at advanced stages, resulting in limited treatment options and poor prognoses. Therefore, early detection of cancer is crucial for improving treatment outcomes and reducing mortality rates (Crosby et al., 2022; Fitzgerald et al., 2022). Current imaging techniques, such as computed tomography (CT), magnetic resonance imaging (MRI), ultrasound imaging and positron emission tomography-computed tomography (PET/CT), utilize contrast agents to improve image contrast and tumor detectability. Nonetheless, these agents may occasionally fail to provide optimal contrast to detect early-stage tumors. Moreover, PET/CT is not considered a routine screening procedure for patients due to radiation exposure and high cost (Bos et al., 2023). Therefore, it is necessary to design more accurate contrast agents and develop new imaging techniques.

Manganese-based contrast agents initially garnered attention due to their bright MRI signal and excellent biocompatibility. Manganese -based contrast agents are considered as ideal alternatives to gadolinium (Gd^3+^) MRI contrast agents. There are two main categories of manganese-based contrast agents: Mn^2+^ complexes and manganese-based nanomaterials (MnNMs). However, Mn^2+^ complexes have a short blood circulation time, leading to their accumulation in the brain and resulting in central nervous system abnormalities (Takeda, 2003; Fitsanakis et al., 2006; Guilarte, 2013; Hu et al., 2014). Therefore, Mn^2+^ complexes are not suitable as MRI contrast agents. In recent years, MnNMs have been discovered to exhibit good T1-weighted contrast effects and negligible toxicity (Pan et al., 2008; Pan et al., 2011). Consequently, MnNMs have been extensively researched as MRI contrast agents. Through clever synthetic design, researchers have also discovered additional imaging capabilities of MnNMs, such as photoacoustic imaging (PAI) (Yan et al., 2023), near infrared fluorescence imaging (NIR-FL) (Li et al., 2022a), and multimodal imaging (Rosenkrans et al., 2021).

Furthermore, MnNMs exhibit an intriguing capability of undergoing degradation within the tumor microenvironment (TME), thereby enabling TME-responsive cargo delivery. This unique property opens up new avenues for utilizing MnNMs in various emerging cancer treatment modalities, including photodynamic therapy (PDT) (Xie et al., 2021), chemodynamic therapy (CDT) (Xin et al., 2021), and sonodynamic therapy (SDT) (Liang et al., 2023). Specifically, MnNMs can be stimulated to decompose endogenous hydrogen peroxide (H_2_O_2_) within the acidic pH conditions prevalent in tumors, leading to the generation of oxygen and Mn^2+^ as reaction byproducts. Moreover, MnNMs exhibit the ability to deplete intracellular glutathione (GSH), thereby augmenting the therapeutic efficacy of PDT, CDT, and SDT. Within the TME, after undergoing a series of chemical reactions, MnNMs can ultimately yield Mn^2+^. Notably, Mn^2+^ serves not only as an MRI contrast agent for enhanced contrast imaging (Gale et al., 2015), but also as a potent stimulator of the cyclic GMP-AMP synthase (cGAS)/stimulator of interferon genes (STING) pathway (Zheng et al., 2023a; Gu et al., 2023; Lei et al., 2024). The cGAS/STING pathway represents an endogenous mechanism within the innate immune system (Zhang et al., 2021), which effectively facilitates immunotherapeutic interventions. In summary, MnNMs represent excellent diagnostic and therapeutic agents for tumor-related applications.

**FIGURE 1:**
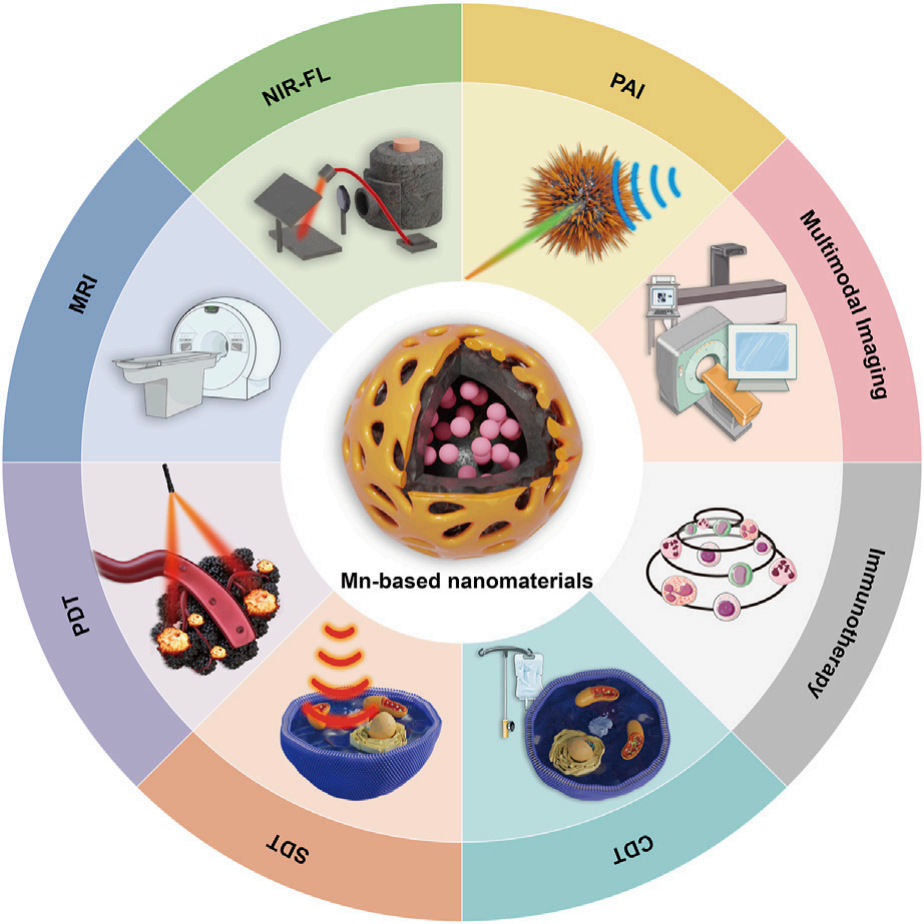
Diagnosis and treatment of cancer mediated by MnNMs.

In this article, we provide a comprehensive overview of recent advancements in cancer research involving MnNMs. We first focus on their applications in tumor diagnosis, with a particular emphasis on their role as contrast agents in MRI due to their inherent paramagnetic properties. Subsequently, we delve into their pivotal role in reshaping the TME for therapeutic purposes, including PDT, CDT, SDT, and immunotherapy (Figure 1).

## 2 The assistance of manganese-based nanomaterials in cancer imaging diagnosis

Medical imaging plays a critical role in the early detection and staging of cancers, as well as in formulating subsequent treatment plans. In recent years, MnNMs have been widely utilized in tumor imaging applications. Initially, owing to their inherent paramagnetic properties, MnNMs were first employed as MRI contrast agents. Subsequently, their utility was expanded to additional modalities including PAI, and NIR-FL. However, individual imaging modalities possess inherent limitations; for example, while MRI provides excellent spatial resolution, it suffers from poor sensitivity. Multimodal imaging integrates multiple complementary imaging techniques into a single platform to overcome the constraints of any single modality (Li et al., 2022b; Sivasubramanian et al., 2022). Through ingenious design, researchers have conferred multimodal imaging capabilities onto MnNMs.

### 2.1 The assistance of manganese-based nanomaterials in MRI

MRI is a radiation-free imaging technique that offers greater penetration depth and high-resolution anatomical images. It has become a crucial clinical tool for early cancer diagnosis (Petralia et al., 2019). To achieve higher imaging contrast, the use of contrast agents is inevitable. Gd^3+^ complexes are commonly employed as contrast agents in MRI (Wahsner et al., 2019; Lu et al., 2022b). However, Gd^3+^ agents may accumulate in the kidneys and brain tissues post-metabolism, posing significant risks to the body (Kanda et al., 2015; Rudnick et al., 2021). This has prompted the search for safer contrast agents. Manganese-based agents were among the earliest reported enhancers for T1-weighted MRI (Niesman et al., 1990). With advancements in nanotechnology, TME responsive manganese nanomaterials have regained attention (Cai et al., 2019; Chen et al., 2022a; Deng et al., 2023). Progress in nanodelivery techniques has led to the emergence of various manganese-based nano-platforms with passive or active targeting capabilities (Patra et al., 2018; Mo et al., 2022). These platforms can respond to the acidic TME, release paramagnetic Mn^2+^, and achieve precise tumor imaging.

**FIGURE 2:**
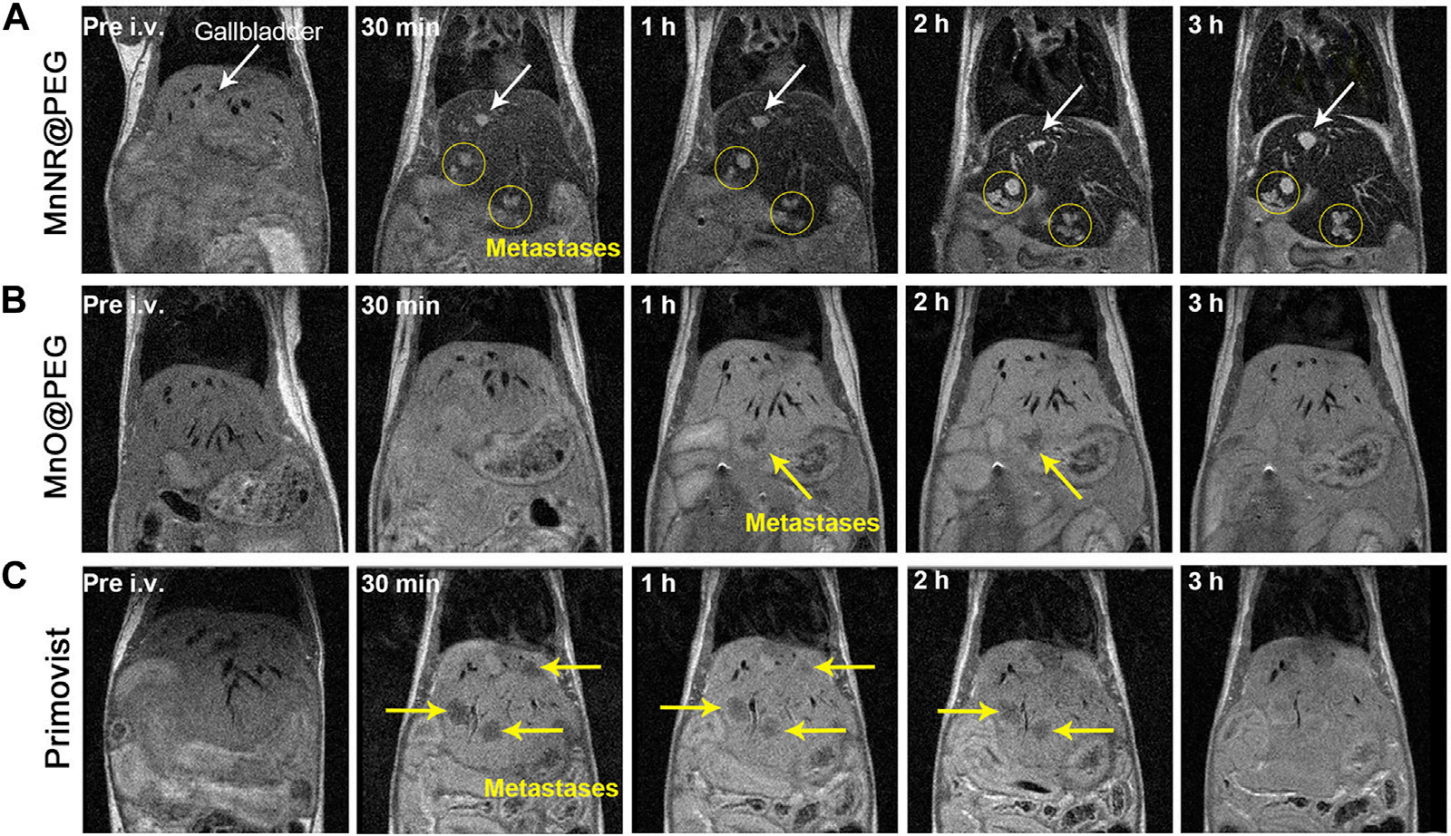
Comparative images at different time points after the injection of three drugs into mice. **(A)** Injection of MnNR@PEG. **(B)** Injection of MnO@PEG. **(C)** Injection of Primovist. (Reprinted from Zhu et al. (2021). Copyright 2021 American Chemical Society).

In 2020, Shi and co-workers synthesized MnCO_3_ nanoparticles using a precipitation method. Further modification with polyethylene glycol (PEG) yielded MnCO_3_ nanorhombuses (MnNRs) (Zhu et al., 2021). MnNRs served as ultra-sensitive T1-weighted MRI contrast agents, exhibiting significant T1 relaxation enhancement in weakly acidic TME conditions. In vivo mouse MRI experiments, these MnCO_3_ nanoparticles selectively highlighted subcutaneous tumors from their periphery to their core. Compared to traditional gadolinium agents Primovist and MnO@PEG, this MnCO_3_ nano-agent enabled high-contrast detection of millimeter-sized liver metastases (Figure 2) (Zhu et al., 2021) and efficient liver excretion through the gallbladder. In subsequent hematoxylin and eosin (H&E) staining and biochemical marker analyses, no evident microscopic lesions were observed. The key biochemical indicators exhibited similarity to the control group, indicating the favorable biocompatibility and low toxicity of MnNRs.

The pH-responsive manganese nanomaterials enable faster and higher tumor imaging sensitivity than clinically used Gd contrast agents. Furthermore, combining manganese nanomaterials with other magnetic metal nanomaterials can further enhance the contrast of MRI (Fan et al., 2021; Wang et al., 2022b; Carregal-Romero et al., 2022). For instance, in a study conducted in 2022, the Tian team developed manganese silicon iron SPIO@SiO_2_@MnO_2_ nanomaterials (Lu et al., 2022a). By comparing them with normal tissue, they observed that in the acidic environment of cancer or inflamed tissue, the MnO_2_ layer decomposed into magnetic-active Mn^2+^ (T1-weighted). They used an imaging processing technique called “dual-contrast enhanced subtraction” to further integrate T1 and T2 contrast differences to enhance imaging sensitivity, enabling the detection of tiny liver metastases.

In addition to responding to the acidic TME, MnNMs can also enhance MRI by reacting with GSH. In 2019, the Hu research team developed hollow manganese/cobalt oxide nanoparticles (MCO NPs) (Ren et al., 2019). These MCO NPs, responsive to GSH, can degrade into Mn^2+^ and Co^2+^, thereby augmenting T1 and T2 weighted MRI contrast. Upon intravenous injection, histological staining images with H&E revealed no significant differences between phosphate buffer solution (PBS) and MCO NPs. This finding substantiates their relative safety.

In summary, carefully designed MnNMs can respond to the TME, decompose, and release Mn^2+^, effectively enhancing MRI signals and improving the efficiency of cancer diagnosis.

### 2.2 The assistance of manganese-based nanomaterials in PAI

PAI is an imaging technology that combines optics and acoustics. It irradiates a target object with pulsed or modulated laser light. The target object absorbs light energy and converts it into heat energy. Target object then undergoes thermal expansion and contraction and radiates sound waves outward. By receiving Acoustic signal, ultrasound detector can achieve image reconstruction of the data (Attia et al., 2019).

PAI contrast agents are substances designed to enhance the contrast of photoacoustic imaging by absorbing light energy and inducing acoustic vibrations in tissues, thereby generating detectable photoacoustic signals. Various contrast agents have been developed for PAI with the aim of improving imaging contrast (Fu et al., 2019). Nanoparticles, including both plasmonic and non-plasmonic types ranging in size from nanometers to hundreds of nanometers, have been employed for this purpose.

**FIGURE 3:**
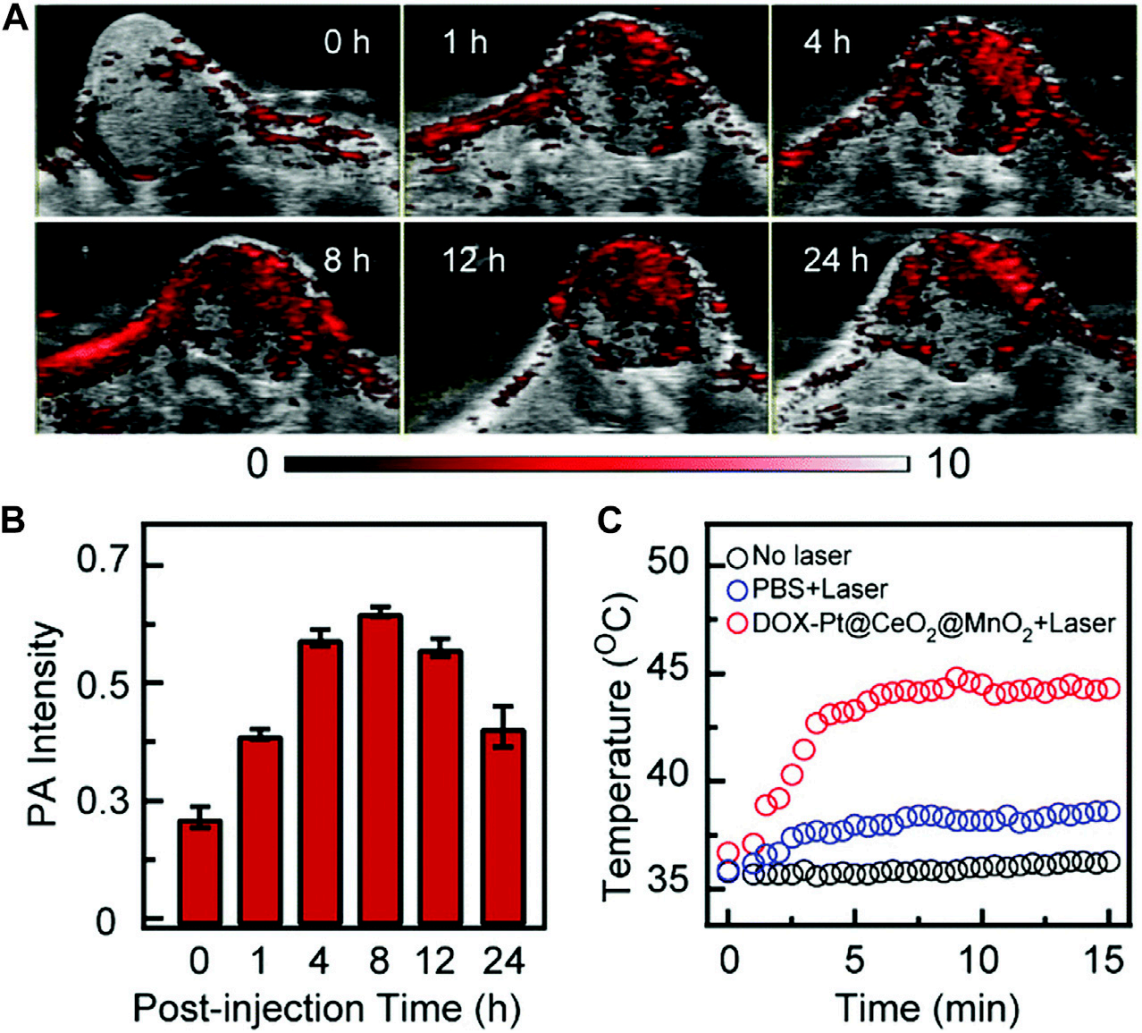
Following the injection of DOX-Pt@CeO_2_@MnO_2_, *in vivo* PAI and temperature alterations in mouse tumors. **(A)** Temporal progression of PAI at the tumor site. **(B)** Quantification of tumor PAI signals over time. **(C)** Changes in surface temperature of the tumor 8 h post-injection of DOX-Pt@CeO_2_@MnO_2_ and PBS, both with and without laser irradiation. (Reprinted from Xu et al. (2021a). Copyright 2021 Nanoscale).

In recent years, MnNMs have been widely studied in the field of PAI due to their responsiveness and excellent light absorption properties (Hu et al., 2019; Huang et al., 2019; Lv et al., 2022). In 2021, the Jiang research team fabricated MnO_2_-coated porous Pt@CeO_2_ core-shell nanostructures (Pt@CeO_2_@MnO_2_) (Xu et al., 2021a). The introduction of MnO_2_ nanomaterials not only imparts responsiveness to the TME to the nanostructure but also enhances light absorption capability significantly. In comparison to Pt NPs and Pt@CeO_2_ nanostructures, the Pt@CeO_2_@MnO_2_ nanostructure exhibits a substantial improvement in light absorption across the ultraviolet to NIR range. Importantly, 24 h post-injection of Pt@CeO_2_@MnO_2_, the photoacoustic intensity in the tumor region remains at 70% of the peak value (Figure 3) (Xu et al., 2021a). Prolonged tumor retention indicates the accumulation of MnO_2_ nanomaterials in the tumor through the enhanced permeability and retention (EPR) effect.

Manganese nanomaterials accumulate at tumor sites through the response to the TME and the EPR effect. Due to their outstanding light absorption properties, manganese nanomaterials effectively enhance PAI signals. This positions manganese nanomaterials as promising substances for PAI contrast agents.

### 2.3 The assistance of manganese-based nanomaterials in NIR-FL

NIR-FL is an imaging technique that exploits light in the NIR region for the excitation and detection of fluorescence signals. By exciting fluorescence signals at the tumor site, effective NIR-FL of tumors can be achieved. Currently, this technology finds extensive applications in the field of tumor surgery (Li et al., 2021). The indispensable component for achieving NIR-FL of tumors is the use of fluorescent probes. Nanomaterials hold a crucial position in NIR-FL due to their optical properties, tunability of surface modifications, and biocompatibility (Jiang et al., 2023).

In NIR-FL, manganese fluorescent nanoprobes can respond to the TME and enhance the effectiveness of tumor imaging. Liu and co-workers developed a hollowed virus bionic MnO_2_ nanoshell, internally loaded with IR1061 and anchored with quantum dots (PbS@CdS) on the surface (Wang et al., 2022c). Upon triggering the MnO_2_ to respond to the TME leading to the degradation and subsequent release of IR1061, precise visualization of tumor margins is achieved. This approach serves the dual purpose of diagnosis and synergistic therapy.

**FIGURE 4:**
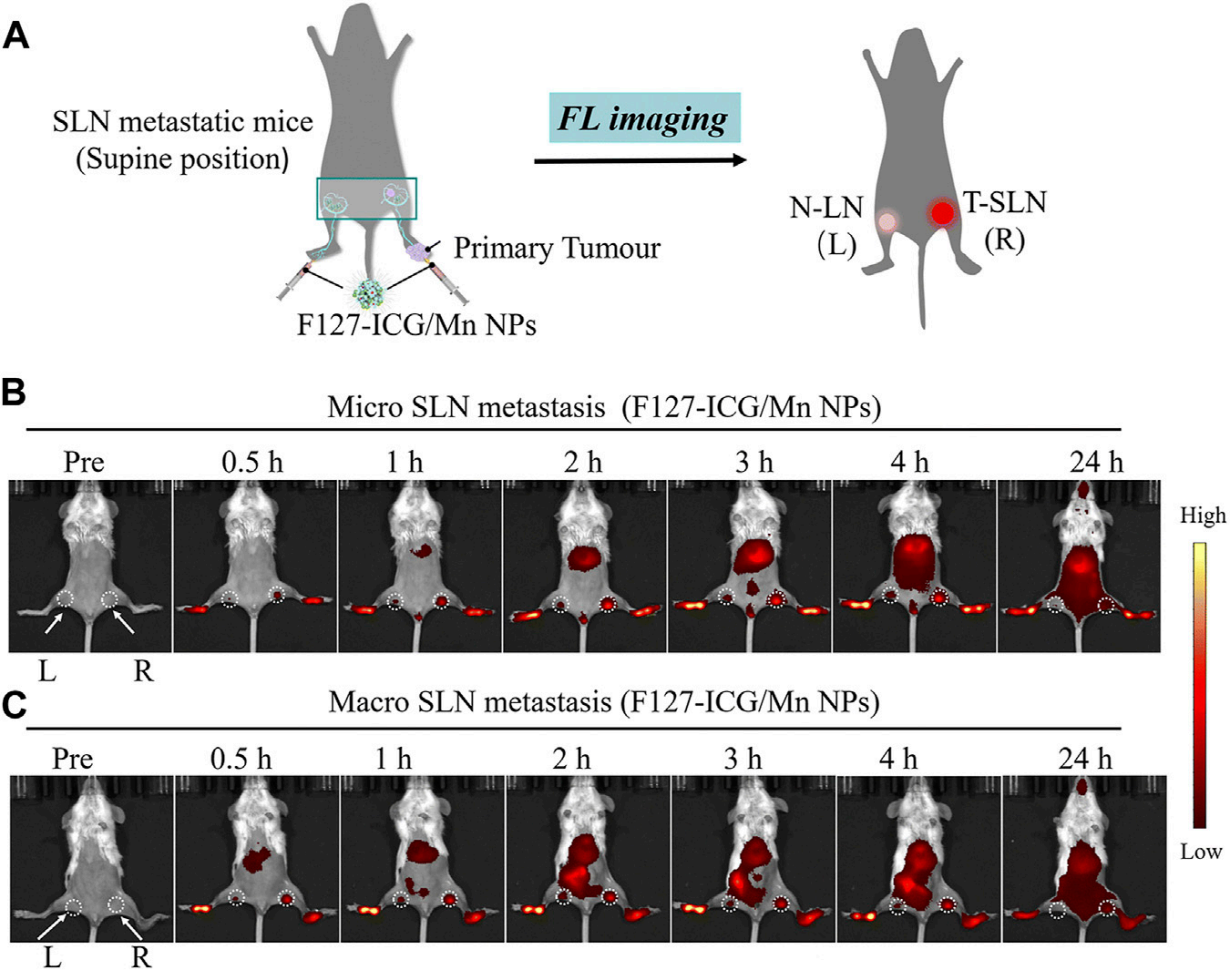
**(A)** Schematic representation of F127-ICG/Mn NPs for NIR-FL. **(B, C)** Images illustrating NIR-FL. (Reprinted from Fu et al. (2022). Copyright 2022 Journal of Materials Chemistry B).

By combining with indocyanine-green (ICG), MnNMs can even enhance the detection of sentinel lymph node metastases associated with tumors. In the study carried out by Ai and co-workers, Manganese porphyrin/ICG nanoparticles were synthesized under the influence of Pluronic F127 surfactant (Fu et al., 2022). Following the subcutaneous injection of this manganese nanomaterial into the footpad of mice, the changes in NIR FL signal intensity were monitored within 24 h. The fluorescence signal intensity variation in the right lymph node (tumor metastatic sentinel lymph node, T-SLN) exceeds that in the left lymph node (normal popliteal lymph node, N-LN). This facilitated a precise differentiation between normal lymph nodes and sentinel lymph nodes linked to tumor metastasis (Figure 4) (Fu et al., 2022).

In summary, MnNMs can be engineered as fluorescent nanoprobes responsive to the TME, facilitating the enhanced release of fluorescent agent and augmentation of NIR-FL. Varied imaging outcomes can be achieved by loading MnNMs with distinct fluorescent substances. These observations underscore the potential of MnNMs in NIR-FL, offering novel insights into cancer diagnostics.

### 2.4 The assistance of manganese-based nanomaterials in multimodal imaging

Stimulus-responsive nanoprobes with integrated multimodal imaging capabilities are highly desirable and dependable for precise tumor visualization. Multimodal imaging methods offer complementary advantages and have gradually become a focus of early cancer screening (Lee et al., 2014). Manganese nanomaterial contrast agents emerge as crucial candidates for MRI contrast agents. Consequently, research on multimodal imaging based on MRI in the field of manganese nanomaterials is continually expanding (Jin et al., 2020; Wen et al., 2022).

**FIGURE 5:**
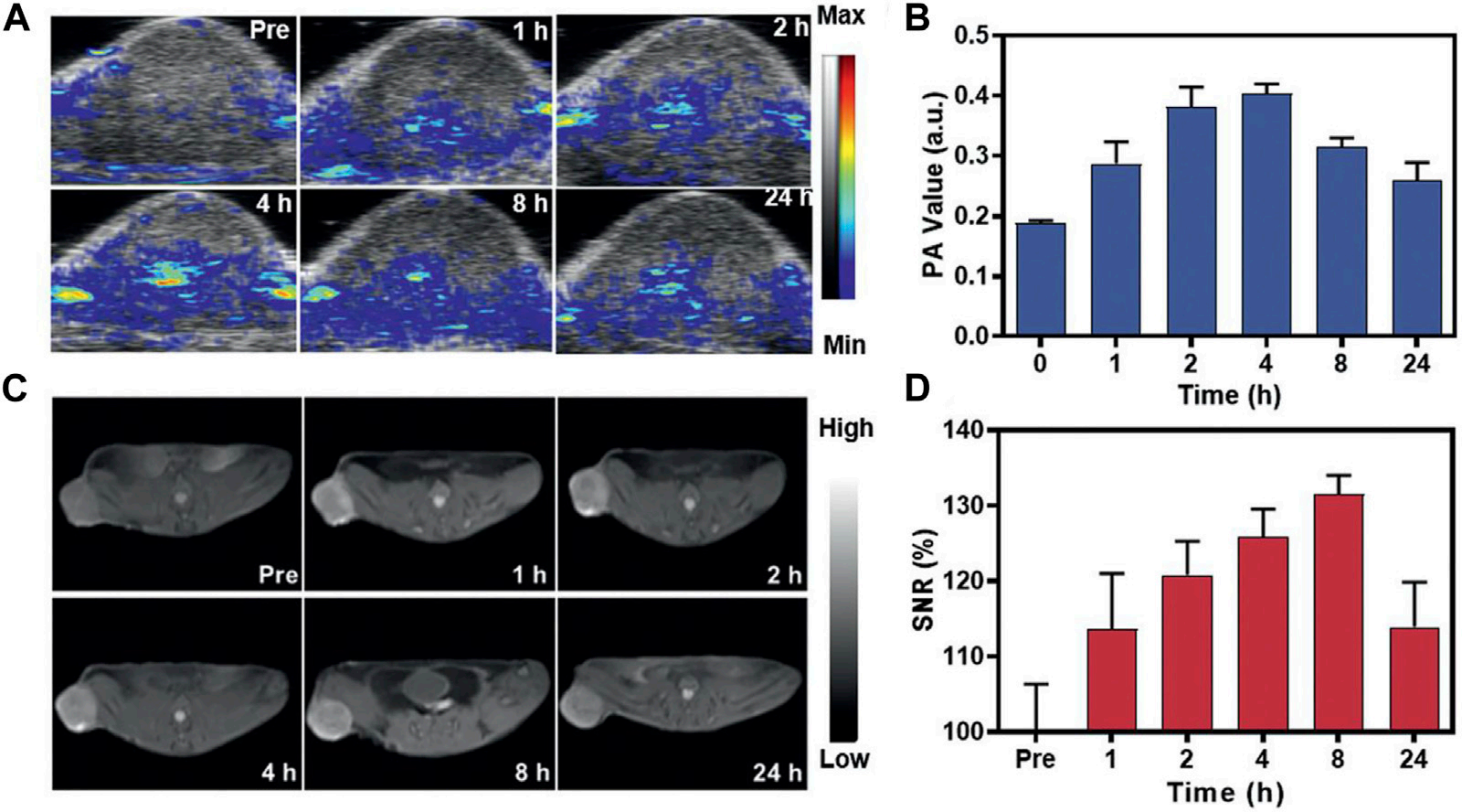
*In vivo* assessment of GSM using PAI and MRI. **(A)** Illustrative PAI images capturing the U87MG tumor at 0, 1, 2, 4, 8, and 24 h after the injection of GSMs (5 mg kg^−1^). **(B)** The corresponding PAI values for the tumors in **(C)**. **(C)** Representative T1-weighted MRI scans of mice administered with GSMs at 0, 1, 2, 4, 8, and 24 h post-injection with GSM. **(D)** Analysis of the signal-to-noise ratio in tumors based on the MRI signals (Reprinted from He et al. (2021). Copyright 2021 Advanced materials).

The integration of PAI and MRI with the use of MnNMs has the potential to significantly enhance tumor diagnostic capabilities. Combining these two modalities enables the simultaneous acquisition of information at both the molecular and tissue structural levels within the same image. Huang and colleagues devised a method involving the plasma modulation of Gold Nanorods (GNRs) through MnO_2_ coating to produce GNR@SiO_2_@MnO_2_ (GSM) (He et al., 2021). A dose of 5 mg kg⁻^1^ of GSM was intravenously administered to mice bearing U87MG tumors, followed by MRI and PAI. The PAI signal from the tumor peaked 4 h after injection, whereas the strongest MRI signal was detected 8 h after injection, indicating a 4-h delay in the MRI signal compared to the PAI signal (Figure 5) (He et al., 2021). This delay could be attributed to the gradual degradation of MnO_2_ in the acidic TME, leading to the release of Mn^2+^ and thereby enhancing the MRI contrast between normal tissue and the tumor.

The combination of MRI and PET is also one of the hot spots in cancer diagnosis research in recent years. Compared to PET-CT, PET-MRI offers advantages such as high image quality, fast scan speeds, and minimal X-ray radiation exposure (Spick et al., 2016). In 2018, a dual-modal imaging probe for PET-MRI was developed by Chen and colleagues (Zhu et al., 2018a). They constructed folic acid-modified multifunctional polyethyleneimine-coated Mn_3_O_4_ nanoparticles, which were subsequently labeled with the radioactive isotope ^64^Cu. This probe demonstrated excellent *in vivo* targeted PET imaging for tumors overexpressing the folate receptor, accompanied by efficient T1-weighted MRI images. The MRI signal intensity enhances with an increase in the concentration of Mn_3_O_4_ nanoparticles.

The combination of MRI and NIR-FL is also widely recognized and studied. Wang and their team engineered a hollow mesoporous manganese-doped, DOX-loaded SiO_2_ shell (Mn-ZGOCS-PEG) (Zou et al., 2021). Mn-ZGOCS-PEG generates Mn^2+^ in response to reductive and acidic TME, enhancing MRI and achieving clear differentiation between muscle and tumor tissues. Over time, as Mn-ZGOCS-PEG degrades within the tumor, the NIR-FL signal at the tumor site gradually strengthens, reaching stability at 60 min, with a sustained NIR-FL signal observed in the tumor area at 180 min.

Integrating multiple imaging modalities for comprehensive tumor visualization is a key goal in cancer treatment, aiming for higher precision and personalization. The paramagnetic properties and tumor-targeting specificity of MnNMs make them highly promising in the field of cancer multimodal imaging diagnosis.

## 3 The assistance of manganese-based nanomaterials in cancer treatment

In addition to common cancer treatment methods like chemotherapy, surgical resection, and radiation therapy, researchers have also pioneered a spectrum of innovative nanomaterial-mediated approaches for cancer treatment, including PDT, SDT, CDT, and immunotherapy (Wu et al., 2022; Kang and Li, 2023). These approaches are at the forefront of cancer research due to their non-invasive nature, targeting capabilities, and potential advantages. MnNMs have gained significant attention in the field of immunotherapy research due to their promising features, including their ability to carry drugs (Xu et al., 2023), modulate the TME (Ding et al., 2021), and activate the cGAS/STING pathway (Wang et al., 2018; Sun et al., 2021). In this section, we will discuss in detail the applications of MnNMs in the context of PDT, SDT, CDT, and immunotherapy for cancer treatment.

### 3.1 The assistance of manganese-based nanomaterials in PDT

PDT is a cancer treatment that utilizes visible light, near-infrared light, or ultraviolet light as an excitation source (Li et al., 2020). Under light exposure, tumor cells can undergo phototoxic cell death induced by light-sensitive materials generating toxic reactive oxygen species (ROS). Currently, a great number of research is focused on MnNMs due to their ability to improve the hypoxic TME and facilitate photosensitizer delivery in PDT (Xu et al., 2021c; Cheng et al., 2022).

**FIGURE 6:**
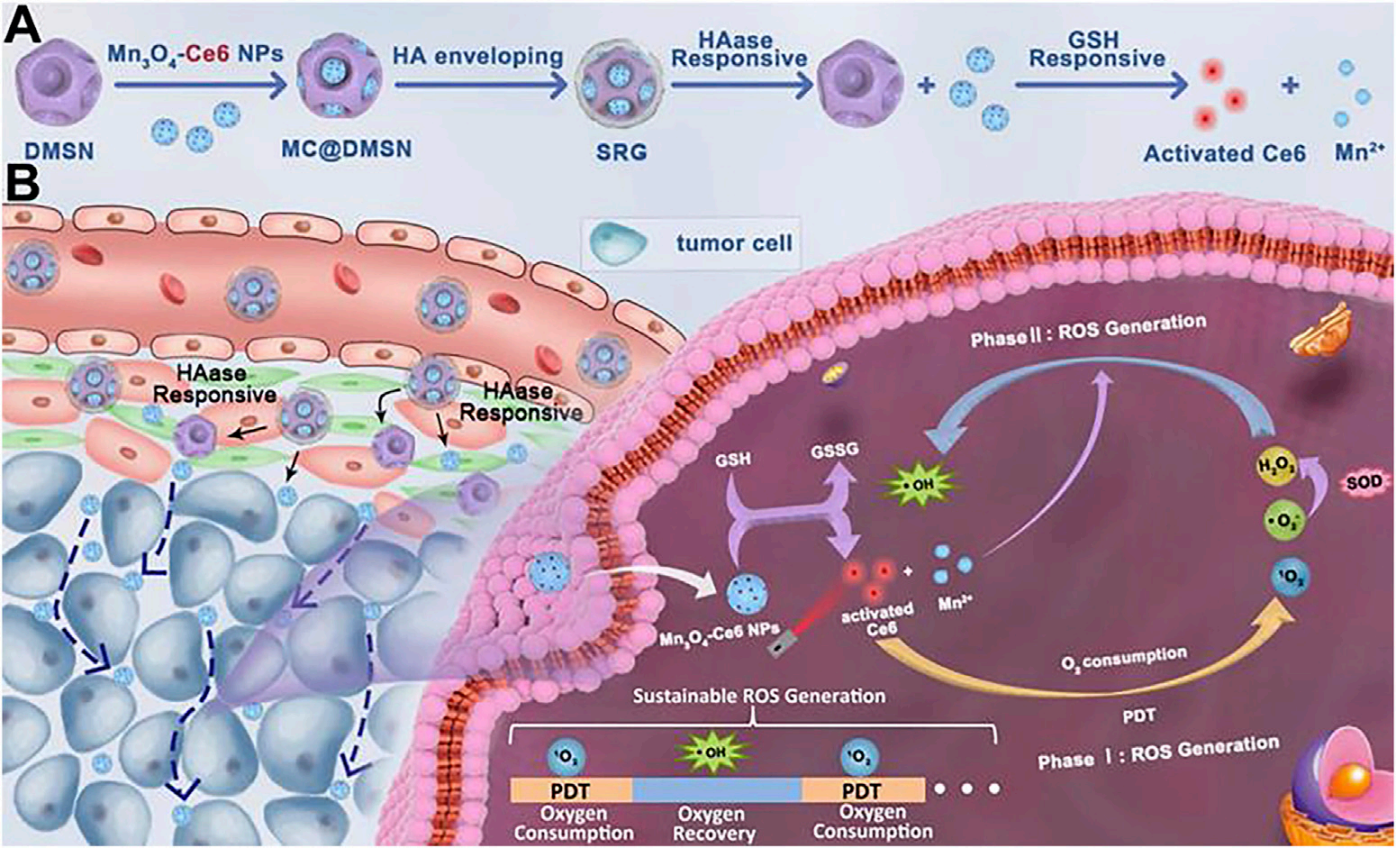
**(A)** After encapsulation by dendritic mesoporous silica nanoparticles (DMSNs), MC is enveloped in hyaluronic acid to form the “Sustainable ROS Generator” (SRG). Under the action of hyaluronidase (HAase), SRG degrades to produce MC. **(B)** *In vivo* behavior of SRG. (Reprinted from Liu et al. (2021). Copyright 2021 Theranostics).

In 2021, Zhang and colleagues devised an innovative strategy to tackle the issue of low levels of ROS in the TME (Figure 6) (Liu et al., 2021). They encapsulated small-sized Mn_3_O_4_-Ce6 nanoparticles (MC) within dendritic mesoporous SiO_2_ nanoparticles and subsequently coated them with hyaluronic acid to create a sustainable ROS generator. This nanomaterial could be evenly distributed throughout the entire tumor. In reaction to the TME, MC undergoes degradation, producing Mn^2+^ that facilitates the sustained transformation of H_2_O_2_ generated during PDT into the highly deleterious ROS. This process intensifies the cytotoxic effects on the tumor. For their study, they selected 4T1 mouse breast cancer cells expressing high levels of GSH as the tumor model cells. They also utilized Hs578Bst human normal breast cells with low GSH expression as control cells to confirm the higher tumor selectivity of Mn_3_O_4_-Ce6 nanoparticles. The outcomes demonstrated a substantial decrease in both tumor volume and weight in the group treated with MnNMs. Moreover, the tumor tissues displayed more extensive damage when compared to other treatment groups, as evidenced by H&E staining.

Combining manganese nanomaterials with suitable metal materials can provide higher photothermal conversion efficiency. In 2020, Zhu et al. reported manganese nanomaterials BSA-Ce6@IrO_2_/MnO_2_ with a remarkable photothermal conversion rate of up to 65.3% (Wu et al., 2020). Ce6 serves as a photosensitizer, while IrO_2_ and MnO_2_ act as catalysts to improve the TME, decompose endogenous H_2_O_2_ to generate oxygen (O_2_), thereby enhancing the efficacy of PDT. Simultaneously, the released Mn^2+^ from the composite material can serve as a contrast agent for MRI.

In general, MnNMs demonstrate promising applications in PDT by improving the hypoxic TME and enhancing the generation of ROS. Furthermore, their combination with other suitable metal materials and photosensitizers may yield additional surprising performances.

### 3.2 The assistance of manganese-based nanomaterials in CDT

CDT, based on Fenton or Fenton-like reactions, transforms H_2_O_2_ into highly toxic hydroxyl radical (•OH) to selectively eliminate tumor cells (Jana and Zhao, 2022). This method was first introduced by Bu, Shi, and their team in 2016 (Zhang et al., 2016). Nevertheless, the high levels of antioxidants in the TME, including GSH, have impeded the clinical translation of this strategy. These antioxidants scavenge ROS to maintain cellular redox homeostasis, thereby substantially reducing the effectiveness of CDT (Cheung and Vousden, 2022). Fortunately, MnNMs possess a strong ability to deplete intracellular antioxidants like GSH. They preferentially accumulate in tumor sites due to EPR, utilizing Fenton-like reactions (Gu et al., 2021; Gao et al., 2022), thereby annihilating tumors.

In 2022, the Liu research team utilized the microemulsion method to synthesize manganese-doped Prussian blue nanoparticles (MnPB NPs) (Tao et al., 2022). Due to the incorporation of Mn^2+^, MnPB NPs demonstrated robust catalytic activity, efficiently converting H_2_O_2_ into •OH through the Fenton reaction. Experimental findings from both *in vitro* and *in vivo* studies indicated that MnPB NPs-mediated CDT exhibited excellent tumor-killing efficacy. MnNMs can synergistically enhance CDT in conjunction with other metallic nanomaterials.

**FIGURE 7:**
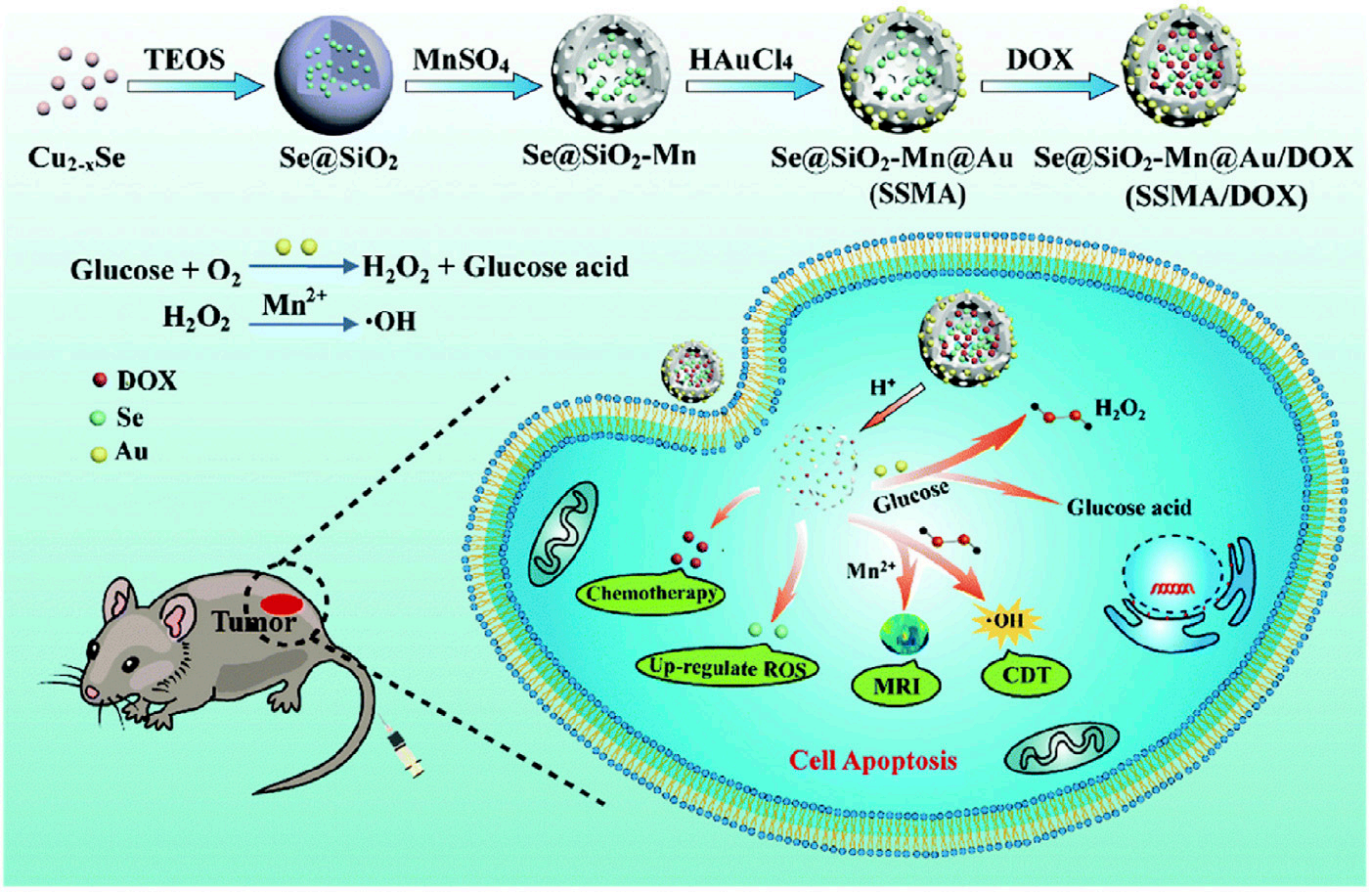
Preparation of SSMA/DOX and schematic diagram of MRI-guided CDT collaborative therapy. (Reprinted from Zheng et al. (2022). Copyright 2022 Journal of Materials Chemistry B).

In another study, the research team led by Hu developed a versatile biomimetic nanozyme, Se@SiO_2_-Mn@Au/DOX (SSMA/DOX) (Zheng et al., 2022). This nanozyme exhibits responsive degradation in the acidic TME, producing Mn^2+^ as a byproduct. Mn^2+^ not only enables therapeutic monitoring through MRI imaging but also catalyzes the conversion of endogenous H_2_O_2_ into •OH for CDT (Figure 7) (Zheng et al., 2022). Additionally, Au NPs catalyze glucose to provide the required H_2_O_2_ for CDT.

In summary, manganese nanomaterials lay the foundation for enhancing CDT by degrading within the TME to generate Mn^2+^, thereby initiating Fenton-like reactions. The integration of manganese nanomaterials with other substances or treatment modalities offers expanded possibilities for their synergistic effectiveness.

### 3.3 The assistance of manganese-based nanomaterials in SDT

SDT is a therapeutic approach that employs low-intensity ultrasound to stimulate sonosensitizers, inducing the generation of reactive oxygen species within tumor cells, thereby leading to the destruction of tumor cells (Pan et al., 2018). It was first introduced by Umemura and colleagues in 1990 (Umemura et al., 1990; Yumita et al., 1990). The selection of suitable sonosensitizers is essential for the efficacy of SDT (Son et al., 2020). Manganese-based nanoscale sonosensitizers, in comparison to traditional organic counterparts, the capability to catalyze H_2_O_2_ molecules for the conversion into O_2_ is instrumental in enhancing SDT.

Li et al. have fabricated nanostructured materials by loading manganese oxide (MnOx) onto piezoelectric bismuth oxychloride nanosheets (BiOCl NS), resulting in M-BOC@SP NSs. The piezotronic effect of BiOCl NS serves as a sound sensitizer. Leveraging the diverse enzymatic-like activities of MnOx, M-BOC@SP NSs not only downregulate the levels of GSH in the TME but also facilitate the decomposition of intracellular H_2_O_2_ into O_2_ and •OH. This process stimulates the production of ROS and reverses hypoxia, thereby enhancing SDT.

**FIGURE 8:**
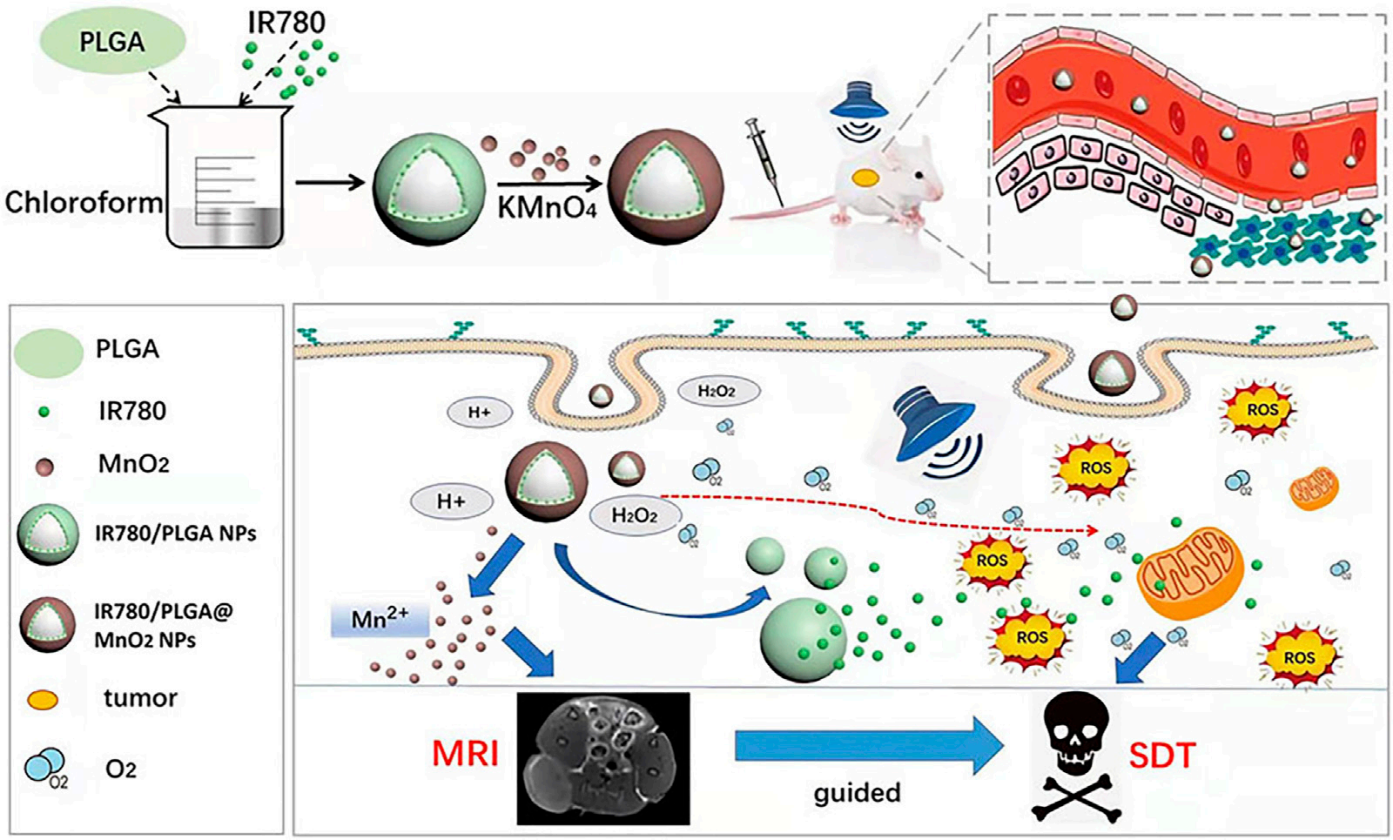
Schematic representation of IR780/PLGA@MnO_2_ NPs employed to enhance breast cancer SDT and MRI (Reprinted from Xu et al. (2022). Copyright 2022 Frontiers in Bioengineering and Biotechnology).

The combination of manganese nanoparticle with organic sonosensitizers also can enhances the efficacy of SDT (Zhu et al., 2018b; Chen et al., 2022b). In 2022, the Niu research team developed IR780/poly (lactide-co-glycolide) (PLGA)@MnO_2_ nanomaterials (Xu et al., 2022). IR780 served as the sonosensitizer, and PLGA was employed to enhance the biocompatibility and stability of IR780. The MnO_2_ nanocoating not only prevented the premature release of IR780 in the bloodstream, enhancing the stability of IR780/PLGA nanomaterials, but also responded to the acidic TME, degrading in acidic conditions to produce O_2_. Upon degradation of MnO_2_, IR780 is released in the tumor, promoting the generation of ROS and enhancing SDT (Figure 8) (Xu et al., 2022). And mn^2+^ can enhance the signal intensity of MRI.

MnNMs primarily enhance SDT by catalyzing the decomposition of H_2_O_2_ into O_2_ within the tumor. When combined with suitable sonosensitizers, MnNMs can also elicit additional effects, such as enhancing ferroptosis and inducing immunogenic cell death (Xu et al., 2021b; Chen et al., 2021). This demonstrates the great potential of manganese nanomaterials in sonodynamic cancer therapy.

### 3.4 The assistance of manganese-based nanomaterials in immunotherapy

In recent years, there has been rapid advancement in the field of immunetherapy, which stands as a potent therapeutic modality for cancer (Dagher et al., 2023). Unlike traditional methods such as surgery, chemotherapy, radiation therapy, and targeted treatment, immunotherapy works by stimulating and enhancing the inherent anti-tumor immune functions to suppress and eliminate cancer cells (Yang et al., 2023). Through the combination with nanomaterials, immunotherapy presents the potential for personalized and precise cancer treatment strategies (Martin et al., 2020).

Similar to most nanomaterials, MnNMs can serve as carriers for delivering immunotherapeutic agents, preventing premature degradation of the immunotherapeutic agents. In 2022, the Shen research team engineered TME-responsive nanomaterials by employing MnO_2_-albumin as a drug carrier, loaded with the PD-L1 inhibitor Butformin (Bu) and the PD-1 inhibitor Methylene Blue (MB), resulting in the preparation of the MB@Bu@MnO_2_ nanomaterial (Zhou et al., 2022). The MnO_2_ nanomaterial selectively delivers the drugs, preventing premature release of MB and Bu. Upon reaching the tumor site, the acidic TME triggers the degradation of MnO_2_, leading to rapid drug release. MnO_2_-mediated O_2_ generation further enhances PDT, subsequently downregulating PD-L1 expression and inhibiting PD-1 activation.

MnNMs also play a role in enhancing anti-tumor immune responses by improving the hypoxic TME (Liang et al., 2018; Luo et al., 2023). In 2021, M. Adjei and colleagues encapsulated MnO_2_ in PLGA to create PLGA-MnO_2_NPs (Murphy et al., 2021). Within the tumor, PLGA-MnO_2_NPs catalyze the generation of oxygen from H_2_O_2_, leading to an improvement in the function of NK cells due to the amelioration of the hypoxic microenvironment. In the situation of PLGA-MnO_2_ NP-induced changes in the TME, NK cells effectively enhance IFN-γ production, and the heightened cytotoxicity against tumor cells is confirmed through Lactate dehydrogenase assay. This suggests that PLGA-MnO_2_NPs can promote tumor immunotherapy by facilitating oxygen production.

In recent years, extensive research has indicated that Mn^2+^ can serve as a cGAS-STING agonist, enhancing tumor immunotherapy (Lv et al., 2020; Wang et al., 2022a). Therefore, MnNMs have garnered increasing attention in the field of tumor immunotherapy (Zheng et al., 2023b; Cheng et al., 2023).

**FIGURE 9:**
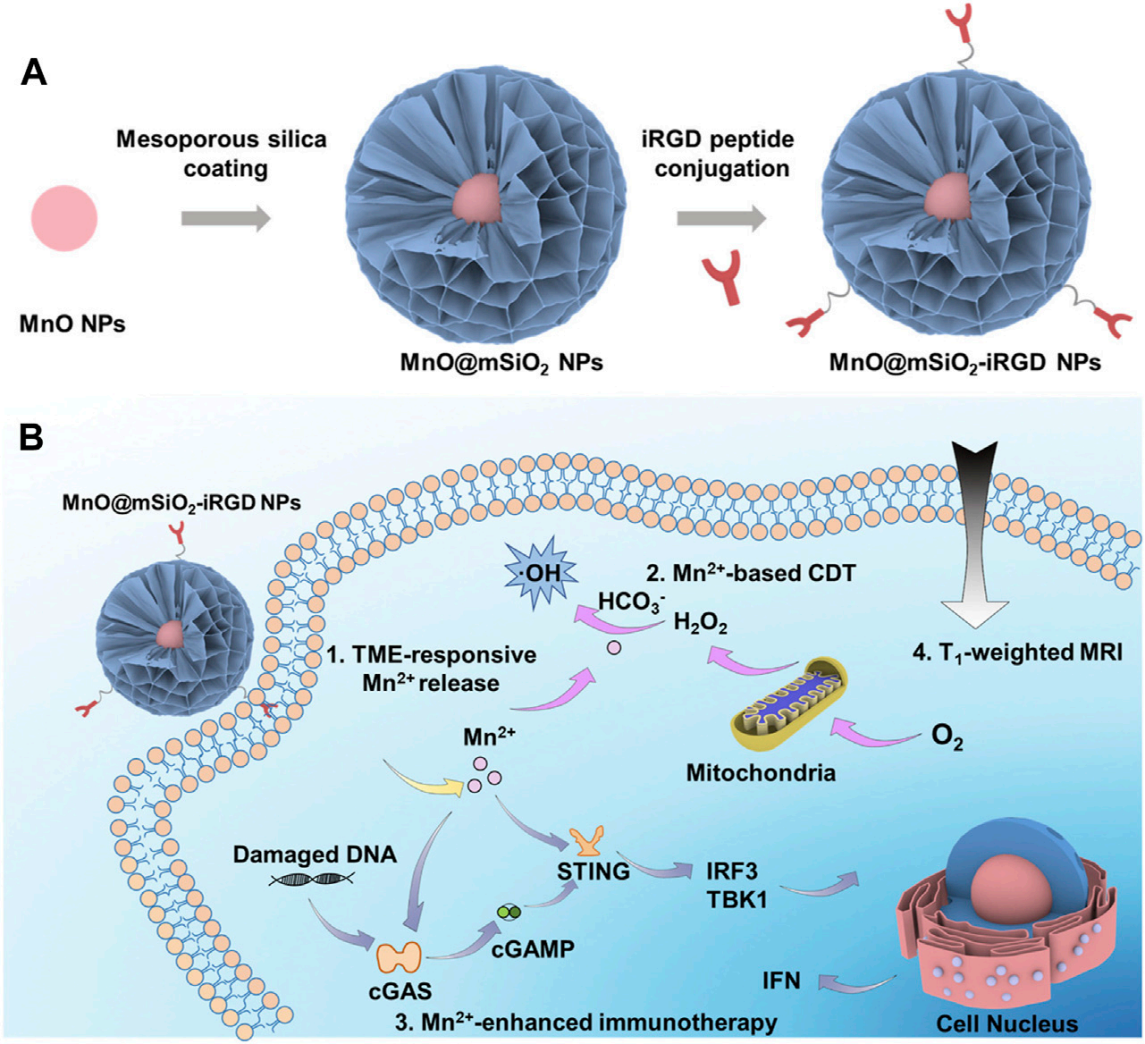
Schematic representation of the synthesis process and theranostic mechanisms of MnO@mSiO_2_-iRGD NPs. **(A)** Depiction of the synthetic process of MnO@mSiO_2_-iRGD NPs. **(B)** Presentation of the mechanisms of MnO@mSiO_2_-iRGD NPs for T1-weighted MRI-guided tumor immune-chemodynamic therapy. (Reprinted from Sun et al. (2022). Copyright 2022 ACS Nano).

In 2022, Hou et al. combined hollow mesoporous SiO_2_-coated MnO nanoparticles with the tumor homing peptide iRGD, constructing MnO@mSiO_2_-iRGD NPs (Sun et al., 2022). MnO@mSiO_2_-iRGD NPs accumulate in tumors through active targeting facilitated by iRGD and respond to the acidic TME, resulting in the decomposition of MnO and the generation of Mn^2+^, enhancing T1-weighted MRI. Upon injecting MnO@mSiO_2_-iRGD nanomaterials into mice, a noticeable upregulation of STING was observed (Figure 9) (Sun et al., 2022). Furthermore, when MnO@mSiO_2_-iRGD and α-PD-1 antibody were used in combination for tumor treatment, the number of CD8^+^ T cells in the tumor tissue significantly increased compared to the use of α-PD-1 antibody alone.

In conclusion, manganese nanostructures can enhance tumor immunotherapy by delivering immunotherapeutic drugs and modulating the tumor immune microenvironment to promote immune responses. Furthermore, the degradation of manganese nanostructures in response to the TME, leading to the release of Mn^2+^ and activation of the cGAS-STING pathway, is being extensively researched as a mechanism to trigger tumor immunotherapy.

## 4 Discussion

In summary, with the continuous increase in cancer incidence, the ongoing development of diagnostic and therapeutic methods is imperative, with nanotechnology playing a crucial role. Therefore, researchers are extensively exploring multifunctional nanomaterial systems. It is noteworthy that MnNMs, owing to their paramagnetic properties and responsiveness to the TME, are poised to play a pivotal role in future imaging diagnostics.

The application of manganese in cancer treatment is noteworthy due to its roles in TME response and modulation. •OH, and O_2_ are generated for cancer therapy through a Fenton-like catalytic reaction between MnOx nanoparticles and H_2_O_2_, effectively improving the hypoxic TME. Hollow MnNMs, functioning as drug carriers, offer promising potential in targeted drug delivery. Furthermore, the degradation of MnNMs within the TME produces Mn^2+^ ions and activates the cGAS-STING pathway, providing evidence for their application in immunotherapy.

Anticipating the future, further research and development in the integration of innovative cancer diagnostic and therapeutic methods with manganese nanomaterials hold the promise of delivering more effective and personalized treatment strategies for cancer patients. However, the majority of current experiments are in the pre-clinical research stage, posing a challenge in expediting their practical application in clinical settings. This challenge encompasses several aspects, including:

1. Biocompatibility and Safety:

While numerous studies have underscored the favorable biocompatibility and low toxicity of manganese-based nanomaterials, several additional factors require careful consideration prior to clinical translation. These factors include distribution, metabolism, potential immune reactions, among others. Currently, the assessment of the biological safety of manganese-based nanomaterials heavily relies on *in vitro* cell viability tests. To expedite clinical translation, there is a pressing need for a systematic and comprehensive collection of substantial and reliable data pertaining to biosafety.

2. Exploring Novel, Efficient Synthesis Pathways:

Despite extensive research into manganese nanoplatform synthesis methods conducted over the past decade, the necessity to explore new, efficient, and direct synthesis routes persists. While progress has been made in comprehending these synthesis pathways, further investigation is imperative to identify simpler and more effective methods of producing these platforms.

3. Integration of Functional Components:

The judicious utilization of various components’ functionalities and their seamless integration into manganese-based nanomaterials to achieve optimal integrated cancer diagnosis and treatment effects poses a significant challenge. Designing these platforms demands meticulous consideration of how different components interact and behave within the TME.

Nevertheless, it is indisputable that significant breakthroughs have been made in the field of biomedical cancer diagnosis and treatment using manganese-based nanomaterials, indicating their substantial developmental potential. The expedited clinical applications of these platforms in cancer treatment can be realized through the initiative-taking addressing of challenges and the strengthened integration of fundamental research with clinical practice. The synergy between basic research and clinical approaches is poised to accelerate the utilization of manganese-based nanomaterials in cancer therapy, confronting these challenges head-on. This approach holds the promise of providing patients with more effective and personalized treatment strategies.
